# Local continual reassessment methods for dose finding and optimization in drug-combination trials

**DOI:** 10.1177/09622802231192955

**Published:** 2023-08-18

**Authors:** Jingyi Zhang, Fangrong Yan, Nolan A Wages, Ruitao Lin

**Affiliations:** 1 Research Center of Biostatistics and Computational Pharmacy, 56651 China Pharmaceutical University, Nanjing, China; 2 Department of Biostatistics, Massey Cancer Center, 6889 Virginia Commonwealth University, Richmond, VA , USA; 3 Department of Biostatistics, The University of Texas MD Anderson Cancer Center, Houston, TX USA

**Keywords:** Bayesian adaptive design, dose optimization, drug combination, early-phase clinical trials, local model

## Abstract

Due to the limited sample size and large dose exploration space, obtaining a desirable dose combination is a challenging task in the early development of combination treatments for cancer patients. Most existing designs for optimizing the dose combination are model-based, requiring significant efforts to elicit parameters or prior distributions. Model-based designs also rely on intensive model calibration and may yield unstable performance in the case of model misspecification or sparse data. We propose to employ local, underparameterized models for dose exploration to reduce the hurdle of model calibration and enhance the design robustness. Building upon the framework of the partial ordering continual reassessment method, we develop local data-based continual reassessment method designs for identifying the maximum tolerated dose combination, using toxicity only, and the optimal biological dose combination, using both toxicity and efficacy, respectively. The local data-based continual reassessment method designs only model the local data from neighboring dose combinations. Therefore, they are flexible in estimating the local space and circumventing unstable characterization of the entire dose-exploration surface. Our simulation studies show that our approach has competitive performance compared to widely used methods for finding maximum tolerated dose combination, and it has advantages over existing model-based methods for optimizing optimal biological dose combination.

## 1. Introduction

For treating cancer, the strategy of combination therapy provides an efficient way to increase patients’ responses by inducing drug–drug synergistic treatment effects, targeting multiple sensitive sites and disease-related pathways, and increasing dose intensity without overlapping toxicities.^1^ Finding a desirable dose combination is critically important for the late-stage development of combination treatments. However, early-phase dose finding/optimization for multiple drugs faces several challenges, such as a large dose exploration space, unclear drug–drug interactions, partially unknown toxicity order among some dose combination pairs, and the small-scale nature of early-phase trials.

In the last decade, numerous designs have been proposed to explore the maximum tolerated dose combination (MTDC) for drug combinations. Most of these designs use parametric models to depict the dose–toxicity relationship and use a similar dose exploration strategy to the well-known, single agent-based continual reassessment method (CRM).^2^ Thall et al.^3^ proposed a two-stage design for identifying three pairs of MTDCs by fitting a six-parameter model. Yin and Yuan^4^ employed a copula-type model to account for the synergistic effect between drugs. Braun and Wang^5^ estimated the dose-limiting toxicity (DLT) rate for drug combinations using a Bayesian hierarchical model and explored the dose matrix using an adaptive Bayesian algorithm. Wages et al.^6^ proposed the partial ordering continual reassessment method (POCRM), transforming the dose-combination matrix into one-dimension and determining the treatment of the next cohort based on the CRM and Bayesian model selection. Riviere et al.^7^ proposed a Bayesian dose finding design for drug-combination trials based on the logistic regression. To circumvent a lack of robustness when using parametric models, several non-parametric or model-free designs have also been proposed. Lin and Yin^8^ developed a two-dimensional Bayesian optimal interval (BOIN) design to identify MTDC. Mozgunov et al.^9^ proposed a surface-free design for phase I dual-agent combination trials. Mander and Sweeting^10^ employed a product of independent Beta probabilities escalation (PIPE) strategy to identify the MTDC contour. Clertant et al.^11^ proposed to identify MTDC or the MTDC contour using a semiparametric method.

Due to the small-scale nature of early phase clinical trials, model-based designs attempt to strike a balance between the comprehensiveness of the dose–toxicity model and the robustness of inference. As a result, parsimonious models are generally proposed to capture the dose–toxicity relationship of the entire dose exploration space in an approximate sense. Despite using parsimonious models to enhance the robustness of inference, model-based designs tend to have unstable performance when the model assumption is misspecified or the observed data is sparse.^12^ This issue becomes more profound for designs of drug-combination trials, where the dimension of the dose-exploration space is large. This difficulty is because more parameters are introduced to quantify the effects of drug–drug interactions. Due to the increased model complexity, prior elicitation or model calibration becomes another hurdle that may affect the operating characteristics.

The goal of dose-finding designs rarely is to estimate the entire dose–toxicity relationship.^13^ Instead, the primary objective is to accurately estimate the local region of the target dose by concentrating as many patients on doses close to the target. To this end, CRM uses a simple, underparameterized model (such as the empiric model) to conduct dose finding. Although it may produce a biased estimate for doses far from the target dose, CRM design converges almost surely to MTD.^14^ Following the main idea of CRM, we propose to use local models to stabilize the dose finding procedure. Specifically, we implement POCRM locally in the adjacent region of the current dose, leading to the local data-based CRM (LOCRM). POCRM aims to characterize the entire dose-toxicity surface by specifying a limited number (typically six to eight) of orderings for the dose exploration space. In contrast, LOCRM evaluates up to five neighboring dose combinations, allowing for the use of all possible toxicity orderings within a local region, bypassing the need for preselection in POCRM. Simulation studies demonstrate that LOCRM design is effective in determining the MTDC and compares favorably with other model-based or model-assisted designs.

As a step further, we extended the LOCRM approach to optimize the dose combination based on the toxicity and efficacy simultaneously. In the modern era of precision oncology, the conventional “more-is-better” paradigm that works for chemotherapies is no longer suitable for targeted therapy and immunotherapy.^15,16^ *Project Optimus*, a recent U.S. FDA initiative, also highlights the need for innovative designs that can handle scenarios where “less-is-more.” By considering both toxicity and efficacy at the same time for decision making, finding an optimal biological dose combination (OBDC) that maximizes the risk–benefit tradeoff in a multi-dimensional dose exploration space becomes even more challenging. In addition to the challenges in finding MTDC, other major obstacles for OBDC finding include the lack of flexible and robust models to account for possible plateau dose–efficacy relationships and the difficulty in effectively assigning patients in the dose-exploration space.

Trial designs for single-agent dose optimization are abundant; see references.^17–23^ However, because of the aforementioned challenges, research on dose optimization methods in dose-combination trials is rather limited. Mandrekar et al.^24^ developed a continual ratio model for dose optimization in drug-combination trials. Yuan and Yin^25^ constructed a Bayesian copula-type model for toxicity and a Bayesian hierarchical model for efficacy. Cai et al.^26^ developed a change point model to identify the possible toxicity plateau on higher dose combinations and employed a five-parameter logistic regression for efficacy estimation. Wages and Conaway^27^ proposed a Bayesian adaptive design with the assumption of monotone dose–toxicity and dose–efficacy relationships within single agents. Guo and Li^28^ proposed dose finding designs based on the partial stochastic ordering assumptions. The two-stage design proposed by Shimamura et al.^29^ includes a zone-finding stage to evaluate toxicity on prespecified partitions and a dose-finding stage to explore the efficacy of the dose space. Yada and Hamada^30^ extended the method of Yuan and Yin^25^ using a Bayesian hierarchical model to share information between doses. As shown in the simulation results, many existing drug-combination dose-optimization designs may suffer from robustness problems due to the use of parametric models to quantify the entire dose-exploration space. To address this, this article introduces a LOCRM12 design based on the proposed LOCRM approach for OBDC identification. We utilize LOCRM as the toxicity model and employ the robit regression model^31^ locally for efficacy. The combination of constructing local models for interim decision-making and using the robit regression makes the trial design more robust and easier to execute in practice.

The remaining of this article is organized as follows: In Section 2, we introduce the local method for modeling toxicity and propose the LOCRM design for MTDC identification. In Section 3, we describe the local efficacy model and introduce the trial design for OBDC identification. In Sections 4 and 5, we conduct extensive simulation studies to evaluate the operating characteristics of the LOCRM and LOCRM12. Section 6 provides a brief discussion.

## 2. Dose finding based on toxicity only

### 2.1. Partial toxicity orderings

Assume that an early-phase drug-combination trial is being conducted to determine MTDC for a combination therapy with J dose levels of drug A and K levels of drug B. Let $p_{j,k}$ be the toxicity probability of dose combination (j,k) , j=1,…,J , k=1,…,K . A key assumption that is usually made for the toxicity of the dose-exploration space is the partial toxicity ordering^6^; that is, the toxicity rate increases with the dose of one drug when the other drug’s dose is fixed at a certain level. We restrict the model and interim decision making within the local space, rather than considering the entire space. Specifically, suppose the current dose is (j,k) , its local dose set is A={(j−1,k),(j,k−1),(j,k),(j+1,k),(j,k+1)} . In other words, dose set A contains all the adjacent dose combinations that differ from the current combination (j,k) by one dose level of one drug. Because we only focus on the local space, we can enumerate all possible toxicity orderings of dose combinations in A . Under the partial monotonicity assumption, we have up to I=4 possible toxicity orderings for local dose combinations contained in A :

Ordering $O_{1}$ : $p_{j-1,k}<p_{j,k-1}<p_{j,k}<p_{j+1,k}<p_{j,k+1}$ ;
Ordering $O_{2}$ : $p_{j,k-1}<p_{j-1,k}<p_{j,k}<p_{j+1,k}<p_{j,k+1}$ ;
Ordering $O_{3}$ : $p_{j-1,k}\;ltp_{j,k-1}\;ltp_{j,k}\;ltp_{j,k+1}\;ltp_{j+1,k}$ ;
Ordering $O_{4}$ : $p_{j,k-1}<p_{j-1,k}<p_{j,k}<p_{j,k+1}<p_{j+1,k}$ .

Under a specific ordering $O_{i},i=1,\ldots ,I$ , let $r_{i}(\;j,k)=1,\ldots ,|A|$ denote the rank of the toxicity rate of dose combination (j,k) (from the smallest to the largest) in the local space A , where |A| is the cardinality (i.e. the total number of dose combinations) of A . For example, when the current dose is (j,k) , under $O_{1}$ , we have $r_{1}(\;j-1,k)=1,r_{1}(\;j,k-1)=2,r_{1}(\;j,k)=3$ , $r_{1}(\;j+1,k)=4$ , and $r_{1}(\;j,k+1)=5$ .

As special cases, if the current dose combination is at the edge of the dose-exploration space, that is, j=1 or J , or k=1 or K , |A| will be smaller than five and the number of possible toxicity orderings will be fewer than four. For example, if the current dose is (1,1) , then the candidate dose set is A={(1,1),(1,2),(2,1)} and the two possible toxicity orderings ( I=2 ) are: $O_{1}$ : $p_{1,1}<p_{1,2}<p_{2,1}$ and $O_{2}$ : $p_{1,1}<p_{2,1}<p_{1,2}$ . If the current dose is (J,1) or (1,K) , |A|=3 and there is only one certain ordering under the partial monotonicity assumption. In particular, if the current combination is (J,1) , A={(J,1),(J,2),(J−1,1)} , we have $p_{J-1,1}<p_{J,1}<p_{J,2}$ . Similarly, if current combination is (1,K) , A={(1,K),(2,K),(1,K−1)} , we have $p_{1,K-1}<p_{1,K}<p_{2,K}$ .

### 2.2. Toxicity model

When I≥2 possible toxicity orderings exist in the local dose combination space A , following the ideas of POCRM^6^ and Bayesian model averaging CRM,^12^ we treat each of the I possible orderings as a probability model and estimate the toxicity rates based on the Bayesian model averaging method.^12^ Because the toxicity orders of the local doses are fully specified under model $O_{i},i=1,\ldots ,I$ , the two-dimensional local space can be converted to a one-dimensional searching line. In other words, a variety of single-agent toxicity models could be employed for an ordering $O_{i},i=1,\ldots ,I$ . Here we employ a common toxicity model of CRM. Based on the commonly used single-parameter empiric CRM model, the toxicity probability of dose combination (j,k)∈A is expressed as

$$p_{j,k}^{\left(i\right)}=\pi (r_{i}(\;j,k){)}^{\exp\;\left(a_{i}\right)}$$

where $a_{i}\in (-\infty ,\infty )$ is the unknown parameter associated with ordering $O_{i}$ , and π(l) is the prior guess of the toxicity probability of the l th dose level, that is, π(1)≤⋯≤π(|A|) is the skeleton of CRM. While other toxicity models like the two-parameter logistic model can be also used, research has shown that these more complex models often perform worse than the simpler one-parameter empirical model in accurately determining the correct dose.^32^

Let $y_{j,k}^{T}$ and $n_{j,k}$ be the number of observed toxicities and the number of patients at combination (j,k) , respectively. Based on the local toxicity data $D_{A}^{T}=\{(y_{j,k}^{T},n_{j,k}):(\;j,k)\in A\}$ , the likelihood function corresponding to ordering $O_{i}$ is

$$\begin{array}{rl} & L\left(D_{A}^{T}|a_{i},O_{i}\right)\\ & =\prod_{\left(\;j,k\right)\in A}\left(\begin{array}{c} n_{j,k}\\ y_{j,k}^{T} \end{array}\right){\left(p_{j,k}^{\left(i\right)}\right)}^{y_{j,k}^{T}}{\left(1-p_{j,k}^{\left(i\right)}\right)}^{n_{j,k}-y_{j,k}^{T}}\\ & =\prod_{\left(\;j,k\right)\in A}\left(\begin{array}{c} n_{j,k}\\ y_{j,k}^{T} \end{array}\right){\left\{\pi (r_{i}(\;j,k){)}^{\exp\;(a_{i})}\right\}}^{y_{j,k}^{T}}\\ & \;{\left\{1-\pi (r_{i}(\;j,k){)}^{\exp\;(a_{i})}\right\}}^{n_{j,k}-y_{j,k}^{T}} \end{array}$$

Let $f(a_{i}|O_{i})$ denote the prior distribution of $a_{i}$ specified under $O_{i}$ . As $a_{i}$ takes values from −∞ to ∞ , a normal distribution $N(0,\sigma_{a}^{2})$ with mean 0 and variance $\sigma_{a}^{2}$ is often used for $f(a_{i}|O_{i})$ in the literature, with $\sigma_{a}^{2}$ typically ranging from 1.34 to 4 .^33,12^ Alternatively, another option is using an exponential distribution like Exp (1) for the prior of $\exp\;(a_{i})$ .^6^ Our simulation study has demonstrated that the proposed design is not sensitive to these prior specifications of $a_{i}$ . The posterior distribution of $a_{i}$ under $O_{i}$ can be expressed as

$$f\left(a_{i}|D_{A}^{T},O_{i}\right)=\frac{L\left(D_{A}^{T}|a_{i},O_{i}\right)f\left(a_{i}|O_{i}\right)}{\int_{-\infty }^{\infty }L\left(D_{A}^{T}|a_{i},O_{i}\right)f\left(a_{i}|O_{i}\right)da_{i}}$$

and the posterior mean of the toxicity rate at dose combination (j,k) is given by ${\hat{p}}_{j,k}^{\left(i\right)}=\int_{-\infty }^{\infty }\pi (r_{i}(\;j,k){)}^{\exp\;(a_{i})}f(a_{i}|D_{A}^{T},O_{i})da_{i}.$

The Bayesian model averaging procedure is conducted to average the posterior estimates of multiple models. Denote the prior probability of each model (ordering) being true as $\mathrm{Pr}(O_{i})$ . We choose equal prior model probabilities with $\mathrm{Pr}(O_{1})=\cdots =\mathrm{Pr}(O_{I})=1/I$ . According to Yin and Yuan,^12^ the posterior model probability $\mathrm{Pr}(O_{i}|D_{A}^{T})$ is

$$\mathrm{Pr}\left(O_{i}|D_{A}^{T}\right)=\frac{L\left(D_{A}^{T}|O_{i}\right)\mathrm{Pr}\left(O_{i}\right)}{\sum_{r=1}^{I}L\left(D_{A}^{T}|O_{r}\right)\mathrm{Pr}\left(O_{r}\right)}$$

where $L(D_{A}^{T}|O_{i})=\int_{-\infty }^{\infty }L(D_{A}^{T}|a_{i},O_{i})f(a_{i}|O_{i})da_{i}$ is the marginal likelihood function of $O_{i}$ . The posterior mean of the toxicity probability of dose (j,k) in A is then calculated as a weighted average of the estimates under each model, that is, ${\bar{p}}_{j,k}=\sum_{i=1}^{I}\mathrm{Pr}(O_{i}|D_{A}^{T}){\hat{p}}_{j,k}^{\left(i\right)}.$

### 2.3. Trial design

Suppose the current dose combination is (j,k) . We treat the next cohort of patients at a dose from the local space, $\left(\;j^{*},k^{*}\right)$ , defined as the dose combination in A with the estimated toxicity probability ${\bar{p}}_{j^{*},k^{*}}$ being closest to the target toxicity probability $\phi_{T}$ , that is,

(1) $$(\;j^{*},k^{*})=\underset{(\;j,k)\in A}{\arg\;\min}\;|{\bar{p}}_{j,k}-\phi_{T}|$$

If multiple dose combinations meet the criteria, we select one randomly. When there is no toxicity outcome observed at the beginning of the trial, dose escalation by LOCRM is equivalent to randomly escalating one dose level of one drug with the level of the other drug fixed. This is because we specify all possible toxicity orderings with equal prior probabilities.

During the trial, we add a safety monitoring rule for overdose control. Given a prespecified probability cutoff $c_{T}$ , if $p_{j,k}$ satisfies

(2) $$\mathrm{Pr}(p_{j,k}>\phi_{T}|D_{A}^{T})>c_{T}$$

then dose combination (j,k) and its higher dose combinations are overly toxic and should be eliminated from the trial. In many cases, using $c_{T}=0.95$ provides satisfactory results for overdose control. But $c_{T}$ can be further calibrated by evaluating various values, such as 0.85, 0.90, and 0.95 through simulations. If the lowest dose satisfies the above condition, then all dose combinations are unacceptably toxic, and we should therefore terminate the trial early. If the trial is terminated because of safety, we do not select any dose as MTDC.

If this overdose control rule is never activated, the trial will continue to recruit and treat patients until the maximum sample size is exhausted. The use of the local data as well as local models can stabilize the dose exploration in the drug-combination space, especially when the amount of observed information is limited and the trial has a large dose exploration space. At the end of the trial, we conduct the bivariate isotonic regression^34^ for the matrix of the observed toxicity rates (function *biviso()* in R package *Iso*). By doing so, the observed information can be shared across all explored dose combinations, leading to a non-decreasing matrix of the estimated toxicity rates and thus an efficient estimate of the final MTDC. After exclusion of untried dose combinations, the dose combination whose isotonic estimated toxicity rate is closest to $\phi_{T}$ is selected as MTDC. If multiple dose combinations satisfy the criteria, the one with lower doses is chosen. Instead of using the bivariate isotonic regression, other model-based approaches (such as the logistic regression model) also can be used to estimate the toxicity rates of the entire space.

## 3. Dose optimization based on the toxicity and efficacy

Based on the LOCRM design proposed in Section 2, we further develop a dose optimization design (i.e. a phase I/II design) for dose-combination trials where the dose escalation/de-escalation decisions are made based on the toxicity and efficacy jointly. This trial design employs LOCRM as the toxicity model and an efficacy model utilizing the same local modeling idea (see the following Section 3.1). We name this dose-optimization design LOCRM12.

### 3.1. Efficacy model

If we construct the efficacy model similar to the toxicity model, we need to enumerate all possible efficacy orderings of candidate doses. However, the partial ordering assumption does not necessarily hold for efficacy. The efficacy surface is much more complicated when taking various dose–efficacy relationships into account. Consider the case when the current combination, (j,k) , is the most efficacious dose combination among the local space A . In this case, the other four candidate doses cannot be ordered, leading to a total of 4!=24 possible orderings. If A has five doses, then we have a total of 5×24=120 possible orderings, which is a huge number to conduct the model averaging procedure as described in Section 2.2. Instead, we propose to use a local regression model for efficacy. We model efficacy based on the local nine dose combinations by the robit model^31^ as a simple robust alternative to the logistic and probit models.

Formally, let $q_{j,k}$ be the efficacy rate at combination (j,k) , and denote B=A∪{(j−1,k−1),(j−1,k+1),(j+1,k−1),(j+1,k+1)} , which includes all possible dose combinations that are adjacent to the current combination (j,k) , then the robit model for efficacy is given by

(3) $$F_{v}^{-1}(q_{j,k})=\alpha +\beta_{1}d_{j}^{A}+\beta_{2}d_{k}^{B}+\gamma_{1}{\left(d_{j}^{A}\right)}^{2}+\gamma_{2}{\left(d_{k}^{B}\right)}^{2}$$

for (j,k)∈B , where $F_{v}^{-1}(\cdot )$ is the cumulative distribution function of Student’s t distribution with v degrees of freedom. The incorporation of the quadratic terms, $(d_{j}^{A}{)}^{2}$ and $(d_{k}^{B}{)}^{2}$ , increases the model flexibility. For example, it enable us to capture the non-monotone dose-response surface. Simulation studies have demonstrated (results omitted) that excluding the two quadratic terms can result in poor operating characteristics for the proposed design. As discussed by Liu,^31^ the robit regression model covers a rich class of flexible models for analysis of binary data, including the logistic (when v≈7 ) and probit (when v→∞ ) models as special cases. Note that the efficacy model incorporates more local data, i.e. a larger local area, than the toxicity model because the dose–efficacy surface is more varied and unpredictable. Section 5.3 demonstrates that utilizing slightly more data improves parameter estimation. Specifically, the design utilizing B for the efficacy model outperforms the design based on A . Furthermore, we omit the interaction term ( $d_{j}^{A}d_{k}^{B}$ ) from the efficacy model since its inclusion does not improve the performance of LOCRM12 in identifying OBDC in small-sized studies (simulation results omitted). This finding is consistent with the results by Cai et al.,^26^ Iasonos et al.,^32^ and Mozgunov et al.^35^

Let $\theta =(\alpha ,\beta_{1},\beta_{2},\gamma_{1},\gamma_{2})$ be the vector of parameters that characterizes the efficacy robit model (3). Denote the number of responders at combination (j,k) as $y_{j,k}^{E}$ . Based on the locally observed efficacy data $D_{B}^{E}=\{(y_{j,k}^{E},n_{j,k}):(\;j,k)\in B\}$ , the likelihood function for efficacy can be written as

$$L\left(D_{B}^{E}|\theta \right)=\prod_{\left(\;j,k\right)\in B}\left(\begin{array}{c} n_{j,k}\\ y_{j,k}^{E} \end{array}\right)q_{j,k}^{y_{j,k}^{E}}(1-q_{j,k}{)}^{n_{j,k}-y_{j,k}^{E}}$$

where the expression of $q_{j,k}$ is given by the robit model (3). Let f(θ) denote the prior distribution of θ . Then the posterior distribution of θ is given by

$$f(\theta |D_{B}^{E})=\frac{L\left(D_{B}^{E}|\theta \right)f(\theta )}{\int L\left(D_{B}^{E}|\theta \right)f(\theta )d\theta }$$

The posterior mean of the efficacy probability $q_{j,k}$ , denote as ${\bar{q}}_{j,k}$ , is

$${\bar{q}}_{j,k}=\int_{}^{}F_{v}(\alpha +\beta_{1}d_{j}^{A}+\beta_{2}d_{k}^{B}+\gamma_{1}{\left(d_{j}^{A}\right)}^{2}+\gamma_{2}{\left(d_{k}^{B}\right)}^{2})f(\theta |D_{B}^{E})d\theta$$

Based on our investigation, we recommend the following prior distribution for θ in the efficacy model

$$\begin{array}{rl} & \alpha ∼N(\mu_{\alpha },\sigma_{\alpha }^{2}),\beta_{1},\beta_{2}∼N(\mu_{\beta },\sigma_{\beta }^{2}),\gamma_{1},\gamma_{2}∼N(\mu_{\gamma },\sigma_{\gamma }^{2})\\ & v∼t(0,1,df),df∼Unif(a_{df},b_{df}) \end{array}$$

where $\mu_{\alpha },\sigma_{\alpha }^{2},\mu_{\beta },\sigma_{\beta }^{2},\mu_{\gamma },\sigma_{\gamma }^{2},a_{df},$ and $b_{df}$ are hyperparameters. In general, the elicitation of the hyperparameters depends on the availability of the prior information and also the operating characteristics through calibration. If there is historical data on monotherapy or combination efficacy, the hyperparameters can be determined using the posterior distribution with a carefully inflated variance. Prior information is often limited in phase I studies, so it is recommended to use large values for $\sigma_{\alpha }^{2}$ , $\sigma_{\beta }^{2}$ , and $\sigma_{\gamma }^{2}$ to ensure a non-informative prior on $q_{j,k}$ . Our investigation finds that using $\sigma_{\alpha }^{2}=\sigma_{\beta }^{2}=\sigma_{\gamma }^{2}=1.3$ or 3 result in similar performance for the proposed design. Our design is not affected by the value of $\mu_{\alpha }$ and thus we recommend $\mu_{\alpha }=0$ for general use. A positive value for $\mu_{\beta }$ is recommended to ensure a monotonically increasing dose–response relationship at lower dose combinations. This facilitates exploration of the dose combination space at the beginning of the trial. In the absence of prior information on the dose-efficacy shape, setting $\mu_{\gamma }=0$ can accommodate a wide range of different shapes. Regarding the prior distribution on the degrees of freedom, we generally recommend $\alpha_{df}=2$ and $\beta_{df}=10$ such that the robit model can balance robustness and good approximation to the logistic or probit model.^31^ In practice, specifying hyperparameters in Bayesian dose-finding trials is challenging and requires close collaboration between statisticians and clinical investigators, who should undertake comprehensive simulation investigations and exploit existing resources. A more systematic way for hyperparameter elicitation can be found by Lin et al.,^36^ where the notion of prior effective sample size (PESS) is adopted to elicit an interpretable and relatively non-informative prior. The choices of $\sigma_{\alpha }^{2}=\sigma_{\beta }^{2}=\sigma_{\gamma }^{2}=1.3$ and 3 both result in PESS being less than 2, respectively.

The proposed designs can be extended straightforwardly to explore combinations of more than two drugs. Suppose we aim to treat patients with p drugs. For the LOCRM design, the local space is expanded to include 2p+1 dose combinations. This includes p higher dose combinations obtained by increasing the dose level of one drug while keeping the dose levels of the other p−1 drugs fixed, and p lower dose combinations in parallel. The dose combinations higher or lower than the current one have p! possible orders, respectively. We can enumerate the p!×p! complete orders in the local space and conduct a trial using the proposed design. For instance, when p=3 , this leads to a space of 36 complete orders. If the number of orders becomes too large, we can consider using the idea of POCRM to specify a subset of the complete orders as candidate models. The efficacy model can be easily extended to include more than two drugs by adding linear and quadratic terms for the additional drugs. The extension of the dose space for the efficacy model follows a similar process as the toxicity model. While it is straightforward to apply the proposed designs to explore combinations of more than two drugs, there may be other practical considerations when investigating the combination of multiple drugs in real-world trials. In practice, due to budget limitations and the consideration that many standard treatments have approved or labeled dose levels, it is more practical to vary the dose levels of a maximum of two or three drugs.

### 3.2. Trial design

We employ the same safety control rule as the LOCRM design (Section 2.3). However, to optimize the toxicity–efficacy trade-off and accommodate the situation where a relatively toxic dose may yield much higher efficacy, we recommend a relatively loose cutoff $\phi_{T}$ that may be slightly larger than the value specified for finding MTDC. The LOCRM12 design consists of two stages; see the dose-finding steps in Algorithm 1. The first stage, that is, the startup stage, is a fast escalation stage to collect preliminary data in the dose matrix. As data is sparse at the start of the trial, we make escalation decisions independent of the toxicity and efficacy models, as long as no toxicity event is observed among the initial cohorts of the patients. Once a toxicity event is observed in the most recent cohort during the safety assessment period, the trial seamlessly enters the main stage.

In the second stage (main stage) of LOCRM12, we use the proposed toxicity and efficacy models to inform escalation/de-escalation decisions. Generally, we treat the next cohort of patients at the locally most efficacious and safe dose combination. Proper safety control and balanced patient assignment between doses are also to be considered. Specifically, we follow a two-step rule to decide the locally optimal dose combination for the next cohort of patients. In the first step, we identify a set of safe dose combinations based on the estimated local MTDC $\left(\;j^{*},k^{*}\right)$ . Specifically, let $\tilde{A}$ denote the admissible set that contains the doses with the posterior estimate of the toxicity probability no higher than the estimated probability of dose combination $\left(\;j^{*},k^{*}\right)$ , that is,

(4) $$\tilde{A}=\{(\;j,k):(\;j,k)\in A\;\mathrm{and}\;{\bar{p}}_{j,k}\leq {\bar{p}}_{j^{*},k^{*}}\}$$

In the next step, we identify the locally most efficacious dose combination in $\tilde{A}$ , denoted as $\left(\;j^{†},k^{†}\right)$ , where $\left(\;j^{†},k^{†}\right)$ achieves the highest posterior efficacy rate by model (3), that is, $\left(\;j^{†},k^{†})=\underset{(\;j,k)\in \tilde{A}}{\arg\;\max}\{{\bar{q}}_{j,k}\right\}$ . Rather than using a simple pick-the-winner rule, we want to enhance the exploration–exploitation trade-off by balancing the patient allocation among the admissible set. In particular, if the current most efficacious dose $\left(\;j^{†},k^{†}\right)$ has never been tried or all dose combinations in $\tilde{A}$ have been tested already, we assign the next cohort of patients to the combination $\left(\;j^{†},k^{†}\right)$ . Otherwise, if $\left(\;j^{†},k^{†}\right)$ has been tried while there are some untried dose combinations in $\tilde{A}$ , we further compare the estimated efficacy probability ${\bar{q}}_{j^{†},k^{†}}$ with a sample size-dependent cutoff, $(\frac{N-n}{N}{)}^{z}$ , where N is the maximum sample size of the main stage, n is the number of patients enrolled in main stage, and z is the tuning parameter to control the degree of balance of patient allocation. If ${\bar{q}}_{j^{†},k^{†}}$ is larger than $(\frac{N-n}{N}{)}^{z}$ , we are confident that treating the next cohort of patients at the optimal dose elicited by the efficacy model is more beneficial. Hence, we allocate the next cohort of patients to $\left(\;j^{†},k^{†}\right)$ . Otherwise, we should explore untried doses. A larger value of z means the investigators have more interest in exploring untried doses and a smaller z indicates more intent to treat patients at $\left(\;j^{†},k^{†}\right)$ . To strike a balance between exploration and exploitation, we recommend z=2 . The sensitivity of the design to difference values of z is investigated in Section 5.3.

In addition to the safety stopping rule used in the LOCRM, we also continuously monitor the efficacy of the considered dose combinations. If the posterior efficacy probability satisfies

(5) $$\mathrm{Pr}(q_{j,k}<\phi_{E}|D_{A}^{E})>c_{E}$$

then the combination (j,k) is deemed futile and should be eliminated from the trial. Here, $\phi_{E}$ is a prespecified lower limit on the efficacy rate, and $c_{E}$ is a probability cutoff. It is recommended to use the response rate of the standard of care or the value specified under the null hypothesis in a Simon’s two-stage design as $\phi_{E}$ in practice. To correctly eliminate overly ineffective dose combinations, $c_{E}$ should be set relatively high. A starting value $c_{E}=0.9$ is recommended, with the option to calibrate the values through simulations if needed. For safety monitoring, because LOCRM12 would trade-off toxicity and efficacy and does not always escalate to the MTDC, we suggest a less stringent value for $c_{T}$ , (say 0.85 or 0.90), for LOCRM12. When all doses are either overly toxic or futile, we should early stop the patient enrollment and conclude that the considered dose space is not desirable.

At the end of the trial, we determine OBDC by finding the most efficacious dose combination among a safe dose set, which is the collection of dose combinations that have the isotonically estimated toxicity probability no greater than that of the selected MTDC. For dose optimization trials that focus on balancing the risk–benefit tradeoff, we recommend using a larger value for $\phi_{T}$ compared to trials finding MTDC only in order to accommodate the scenarios where a slightly over-toxic dose combination may yield much higher efficacy than the lower dose combinations. Therefore, the selected MTDC in a dose optimization trial usually is higher than the MTDC picked in a conventional dose-finding trial. The selected OBDC is the dose combination that has been tested in the trial and has the highest estimated efficacy probability among the safe dose set. The excluded dose combinations that are either overly toxic or futile should not be considered as the candidates for OBDC.

## 4. Simulation studies for finding MTDC

To evaluate the operating characteristics of the proposed LOCRM design, we choose a model-based design (POCRM^6^) and a model-assisted design (BOIN ${}_{\mathrm{Comb}}$ ^8^) as competing methods. Six representative scenarios are presented in Table 1. Two MTDCs exist in Scenarios 1 and 6, whereas, three MTDCs exist in Scenarios 2 to 5. The MTDCs are located at the relative lower part of the dose space in Scenario 1, and the MTDCs progress towards higher regions in the dose exploration space as we go from Scenarios 1 to 6. The target toxicity rate is set as $\phi_{T}=0.3$ , and the cutoff value is $c_{T}=0.95$ . The maximum sample size is assumed to be 51 and patients are treated in cohorts of size three. Under each design, we simulate 5000 trials for each scenario.

**Table 1.** True toxicity probabilities of Scenarios 1 to 6 for evaluating the LOCRM design and its competing methods.

	Drug A
Drug B	1	2	3	4	5	1	2	3	4	5
	Scenario 1					Scenario 2				
1	0.15	**0.30**	0.45	0.50	0.60	0.05	0.10	**0.30**	0.45	0.55
2	**0.30**	0.45	0.50	0.60	0.75	0.10	**0.30**	0.45	0.55	0.70
3	0.45	0.55	0.60	0.70	0.80	**0.30**	0.40	0.50	0.60	0.75
	Scenario 3					Scenario 4				
1	0.05	0.10	0.20	**0.30**	0.40	0.05	0.10	0.15	**0.30**	0.45
2	0.10	0.20	**0.30**	0.40	0.55	0.10	0.15	**0.30**	0.45	0.55
3	**0.30**	0.40	0.45	0.50	0.60	0.15	**0.30**	0.45	0.50	0.60
	Scenario 5					Scenario 6				
1	0.02	0.07	0.10	0.15	**0.30**	0.01	0.02	0.08	0.10	0.11
2	0.07	0.10	0.15	**0.30**	0.45	0.03	0.05	0.10	0.13	**0.30**
3	0.10	0.15	**0.30**	0.45	0.55	0.07	0.09	0.12	**0.30**	0.45

LOCRM: local data-based continual reassessment method; MTDCs: maximum tolerated dose combinations.

MTDCs are defined as the dose combinations with a toxicity rate of 0.3 and are highlighted in boldface.

For the LOCRM design, we generate skeletons by using the algorithm proposed by Lee and Cheung^33^ (function *getprior()* in R package *dfcrm*). To implement the *getprior()* function, one needs to prespecify the target rate ( $\phi_{T}=0.3$ ), the number of candidate doses ( =3,4, or 5), the prior guess of MTD ( =2,3, or 4), and the halfwidth of the indifference interval ( =0.05 ). The indifference interval refers to a range of toxicity rates such that a dose level whose toxicity rate falls within this interval can be considered equivalent to the MTD. The prior distribution of parameter $a_{i}$ is set as N(0,2) , i=1,…,I . Assuming six toxicity orderings,^37^ we simulate the POCRM design by modifying R package *pocrm*. Specifically, in the startup stage, we randomly increase the amount of drug A or B and fix the amount of the other drug, and the trial transforms to the next stage when toxicity events are observed. This new startup rule makes the POCRM design more comparable with the other two designs. The skeleton used in POCRM is chosen using the *getprior()* function with the prior MTD guess as the seventh dose and a halfwidth of the indifference interval being 0.05. Results of BOIN ${}_{\mathrm{Comb}}$ are generated by R package *BOIN* using the default configuration. For a fair comparison between the competing methods, we use the same overdose control rule (2) based on the beta-binomial model for all designs.

The performance of LOCRM and other methods is assessed based on four metrics: (a) percentage of trials that ultimately choose one of the true MTDCs; (b) number of patients assigned to any of the true MTDCs; (c) percentage of trials that end up selecting a dose combination with a toxicity rate greater than $\phi_{T}$ ; and (d) number of patients treated at overly toxic doses. The first two metrics evaluate the design’s ability to identify the target dose combinations and high values are desirable. The latter two metrics are used to assess safety, thus smaller values indicate better safety control. The operating characteristics of LOCRM and the competing methods are summarized in Table 2. On average, the proposed LOCRM design is comparable to POCRM and BOIN ${}_{\mathrm{Comb}}$ design in terms of the four performance indicators. The difference of the percentage of selecting true MTDCs is no more than 11 points between different methods under various scenarios. The proposed design exhibits better safety control on average, especially in Scenarios 1 to 4, where the MTDCs are at the middle part of the dose matrix.

**Table 2.** Simulation results of the LOCRM, POCRM, and BOIN ${}_{\mathrm{Comb}}$ designs under the toxicity Scenarios 1 to 6.

Method	MTDC(s)		Overdose(s)	
	Sel%	No. of pts	Sel %	No. of pts
Scenario 1				
LOCRM	73	27	17	11
POCRM	62	22	33	22
BOIN ${}_{\mathrm{Comb}}$	68	25	23	14
Scenario 2				
LOCRM	74	27	19	11
POCRM	68	26	27	20
BOIN ${}_{\mathrm{Comb}}$	69	24	25	14
Scenario 3				
LOCRM	48	15	22	11
POCRM	48	19	31	18
BOIN ${}_{\mathrm{Comb}}$	48	15	31	14
Scenario 4				
LOCRM	65	21	14	8
POCRM	65	23	19	14
BOIN ${}_{\mathrm{Comb}}$	66	20	18	10
Scenario 5				
LOCRM	61	18	13	7
POCRM	67	24	12	9
BOIN ${}_{\mathrm{Comb}}$	64	18	17	9
Scenario 6				
LOCRM	66	17	11	7
POCRM	69	22	9	7
BOIN ${}_{\mathrm{Comb}}$	67	18	13	7

LOCRM: local data-based continual reassessment method; POCRM: partial ordering continual reassessment method; BOIN: Bayesian optimal interval; MTDCs: maximum tolerated dose combinations.

Sel% means the selection percentage of MTDCs or overdose(s). No. of pts means number of patients treated at MTDCs or overdose(s).

**Table 3.** True toxicity and efficacy probabilities of Scenarios 7 to 16 for evaluating the performance of the LOCRM12, Cai, and YY designs.

	Drug A
	1	2	3	4	5	1	2	3	4	5
Drug B	Toxicity probability					Efficacy probability				
	Scenario 7									
1	0.05	0.15	0.30	0.45	0.55	0.05	0.25	**0.50**	0.55	0.60
2	0.15	0.30	0.45	0.55	0.65	0.25	**0.50**	0.55	0.60	0.65
3	0.30	0.45	0.55	0.65	0.75	**0.50**	0.55	0.60	0.65	0.70
	Scenario 8									
1	0.10	0.15	0.21	0.30	0.42	0.10	0.18	0.35	**0.50**	0.52
2	0.15	0.24	0.30	0.42	0.44	0.15	0.35	**0.50**	0.52	0.53
3	0.20	0.30	0.42	0.44	0.51	0.20	**0.50**	0.52	0.54	0.56
	Scenario 9									
1	0.05	0.10	0.18	0.25	0.42	0.30	**0.45**	**0.60**	**0.45**	0.26
2	0.10	0.15	0.23	0.42	0.43	0.20	0.28	**0.45**	0.26	0.18
3	0.15	0.23	0.45	0.50	0.55	0.10	0.14	0.24	0.18	0.10
	Scenario 10									
1	0.02	0.04	0.07	0.12	0.18	0.10	0.30	**0.45**	0.30	0.08
2	0.04	0.08	0.13	0.18	0.25	0.25	**0.45**	**0.60**	**0.45**	0.23
3	0.14	0.25	0.25	0.25	0.25	0.20	0.30	**0.45**	0.30	0.16
	Scenario 11									
1	0.15	0.21	0.30	0.42	0.44	0.20	**0.45**	0.33	0.15	0.05
2	0.24	0.30	0.42	0.44	0.51	0.35	**0.60**	0.45	0.20	0.15
3	0.30	0.33	0.44	0.51	0.55	0.20	**0.45**	0.30	0.15	0.10
	Scenario 12									
1	0.05	0.09	0.17	0.24	0.30	0.20	0.29	**0.55**	0.20	0.15
2	0.15	0.19	0.23	0.35	0.42	0.30	0.39	**0.55**	0.25	0.20
3	0.34	0.38	0.43	0.51	0.66	0.36	0.35	0.30	0.23	0.20
	Scenario 13									
1	0.05	0.09	0.14	0.23	0.30	0.10	0.18	0.25	0.30	0.31
2	0.11	0.15	0.17	0.24	0.42	0.20	0.28	0.35	**0.50**	0.52
3	0.14	0.18	0.23	0.41	0.46	0.23	0.30	**0.50**	0.52	0.53
	Scenario 14									
1	0.10	0.15	0.30	0.35	0.45	0.10	0.20	0.30	**0.50**	0.55
2	0.15	0.20	0.35	0.45	0.50	0.15	0.25	**0.50**	0.55	0.60
3	0.20	0.30	0.35	0.51	0.60	0.20	0.30	**0.50**	0.60	0.70
	Scenario 15									
1	0.15	0.21	0.30	0.42	0.44	0.21	0.15	0.12	0.09	0.05
2	0.24	0.30	0.42	0.44	0.51	**0.60**	**0.45**	0.33	0.24	0.21
3	0.30	0.33	0.44	0.51	0.55	**0.45**	0.31	0.24	0.21	0.17
	Scenario 16									
1	0.50	0.56	0.65	0.68	0.72	0.52	0.62	0.70	0.76	0.79
2	0.55	0.62	0.70	0.72	0.80	0.55	0.66	0.74	0.79	0.82
3	0.60	0.67	0.75	0.79	0.85	0.58	0.70	0.78	0.82	0.85

LOCRM: local data-based continual reassessment method; Cai et al.: Cai; YY: Yuan and Yin; OBDC(s): optimal biological dose combination(s); TDC(s): target dose combination(s). OBDC(s) are in boldface and underline. TDC(s) are in boldface.

## 5. Simulation studies for finding OBDC

### 5.1. Simulation configuration

To demonstrate the desirable performance of the proposed LOCRM12 design, we compare it with two competing methods, Cai et al.^26^ (referred to as Cai) and Yuan and Yin^25^ (referred to as YY). Ten representative scenarios with five doses for drug A and three doses for drug B are present in Table 3. The 10 scenarios incorporate various dose–toxicity and dose–efficacy relationships that can be seen in real trials. The raw doses for drug A and drug B are (0.08,0.16,0.24,0.32,0.40) and (0.08,0.16,0.24) , respectively. In Scenarios 7, 8, 13, and 14, both the dose–toxicity and dose–efficacy relationships are monotone, hence the optimal doses are the highest safe doses. In Scenario 9, the dose–efficacy relationship is unimodal for drug A and monotonically decreasing for drug B, leading to the optimal dose being the one with a moderate dose of drug A and a low dose of drug B. In Scenarios 10 and 11, the OBDCs are located at the middle of the dose matrix as the dose–efficacy relationship is unimodal for both drug A and drug B. In Scenario 15, efficacy is monotonically decreasing with the dose in drug A and unimodal with the dose in drug B. In Scenario 16, all dose combinations are overly toxic and none of the candidate dose combinations should be determined as OBDC.

The upper limit for the toxicity rate is set as $\phi_{T}=0.35$ . Using equation (2) for overdose control, the cutoff value is set as $c_{T}=0.85$ . The parameter specification of the toxicity model used for the LOCRM12 design is the same as that described in Section 4 for the LOCRM design. The lower limit for the efficacy rate is set as $\phi_{E}=0.2$ . Using equation (5) to monitor efficacy, the cutoff value is set as $c_{E}=0.9$ . The hyperparameters in the efficacy model in equation (3) are set as follows: $\mu_{\alpha }=\mu_{\gamma }=0$ , $\mu_{\beta }=0.8$ , $\sigma_{\alpha }^{2}=\sigma_{\beta }^{2}=\sigma_{\gamma }^{2}=1.3,a_{df}=2,b_{df}=10$ . The impact of various prior distributions is investigated in Section 5.3. For the LOCRM12 and Cai designs, we get the standardized doses $d_{A}=(-1.26,-0.63,0,0.63,1.26)$ and $d_{B}=(-1,0,1)$ for drug A and drug B, respectively, such that the standardized doses are centered around mean 0 with a unit standard deviation. We also take z=2 for the LOCRM12 and Cai designs. For the YY design, we set the lowest acceptable response rate as 0.2, consistent with the value of $\phi_{E}$ used in LOCRM12. The number of patients for the first and second stages are 21 and 30, respectively.

### 5.2. Operating characteristics

The evaluation of operating characteristics for the proposed and competing methods are based on the following six indicators: (a) selection percentage of true OBDC(s); (b) number of patients treated at OBDC(s); (c) selection percentage of target dose combinations (TDCs); (d) number of patients treated at TDC(s); (e) selection percentage of overly toxic dose(s); (f) number of patients treated at overly toxic dose(s). Indicators (a) and (b) are to assess the design’s ability to identify optimal dose combinations. Since all doses are overly toxic in Scenario 16, selecting any dose as OBDC is not expected. Thus, we summarize the probability of early stopping and the average number of patients that are not enrolled in the trial. We define TDCs as the dose combinations with a response rate no smaller than 0.45 among the safe dose combinations. As a result, (c) and (d) assess the probability of avoiding treating patients at subtherapeutic levels. Large values of indicators (a) to (d) are preferred. Indicators (e) and (f) are to evaluate the safety profile of the competing designs. Smaller values of the two indicators are preferred.

The operating characteristics of the proposed LOCRM12 design and competing methods are summarized in Table 4. In Scenarios 7 and 8, where OBDCs are located at the off-diagonal of the dose matrix, LOCRM12 performs the best in OBDC identification. In Scenario 7 particularly, LOCRM12 selects the correct OBDC with a probability 7.5% larger than the Cai design (59.0% vs. 51.5%) and 21.4% larger than the YY design (59.0% vs. 37.6%) design. LOCRM12 also allocates 7.0 and 12.9 more patients to OBDC than Cai (24.0 vs. 17.0) and YY (24.0 vs. 11.1), respectively. In terms of safety, the LOCRM12 design exhibits a better safety profile than the Cai design. As observed in Scenarios 7 and 8, the Cai design allocates 9.3 and 15.0 more patients to overly toxic dose combinations, respectively. In Scenarios 9 and 14, LOCRM12 yields comparable performance with the Cai design in terms of OBDC identification, while it shows an advantage in terms of safety control. In Scenarios 10 to 13, the LOCRM12 design is the most efficient for OBDC identification. In Scenario 11, the YY design shows the best safety control, but a limited ability to accurately select the OBDC(s) and TDC(s). In Scenario 15, the YY design performs the best in selecting OBDC and TDCs. However, the LOCRM12 design still possesses an advantage in terms of patient allocation. For example, the LOCRM12 design allocates 5.7 and 11.3 more patients than YY to OBDC and TDCs, respectively. Moreover, the YY design is very sensitive to scenario specifications. For example, in Scenario 13, this design selects OBDC(s) with a probability of 0.7% and selects the overly toxic dose combinations with a probability of 50.6%. In Scenario 16, where all dose combinations are overly toxic, LOCRM12 has similar performance as the Cai and YY designs. In Scenarios 9–11 and 15, where TDCs include more dose combinations than OBDCs, the LOCRM12 design exhibits promising operating characteristics in terms of both the selection percentage of TDCs and the number of patients treated at TDCs. For example, in Scenario 10, the selection percentage of TDCs of the LOCRM12 design is as high as 86.7%, while those of Cai and YY are 81.6% and 3.9%, respectively. The number of patients treated at TDCs based on the LOCRM12, Cai, and YY are 32.2, 29.1, and 4.6, respectively. In Scenarios 9 and 15, the selection percentages of TDCs are comparable between LOCRM12 and Cai, but the LOCRM12 design allocates more patients to TDCs.

**Table 4.** Simulation results of the LOCRM12, Cai, and YY designs.

	OBDC(s)		TDC(s)		Overdoses	
Method	Sel%	No. of pts	Sel%	No. of pts	Sel%	No. of pts
Scenario 7						
LOCRM12	59.0	24.0	59.0	24.0	27.1	14.2
Cai	51.5	17.0	51.5	17.0	35.0	23.5
YY	37.6	11.1	37.6	11.1	19.8	18.7
Scenario 8						
LOCRM12	42.2	16.2	42.2	16.2	19.6	9.5
Cai	34.5	10.8	34.5	10.8	46.5	24.5
YY	16.9	9.4	16.9	9.4	31.1	14.7
Scenario 9						
LOCRM12	42.5	11.4	79.0	28.8	2.6	6.8
Cai	42.3	10.0	79.4	24.5	7.4	11.9
YY	4.9	2.1	16.9	11.4	29.1	17.5
Scenario 10						
LOCRM12	46.8	12.4	86.7	32.2	0.0	0.0
Cai	36.4	9.3	81.6	29.1	0.0	0.0
YY	2.8	1.0	3.9	4.6	0.0	0.0
Scenario 11						
LOCRM12	29.0	11.4	46.0	20.8	8.1	6.2
Cai	24.8	7.7	46.7	16.1	11.3	12.8
YY	15.3	2.6	37.1	10.0	5.1	1.6
Scenario 12						
LOCRM12	45.7	17.1	45.7	17.1	5.8	6.5
Cai	39.8	11.0	39.8	11.0	13.6	12.3
YY	16.9	6.2	16.9	6.2	12.4	10.7
Scenario 13						
LOCRM12	39.1	14.3	39.1	14.3	16.0	8.5
Cai	31.4	11.8	31.4	11.8	49.3	18.9
YY	0.7	1.7	0.7	1.7	50.6	17.0
Scenario 14						
LOCRM12	35.6	14.2	35.6	14.2	8.3	5.0
Cai	36.8	12.8	36.8	12.8	27.2	18.4
YY	13.6	9.5	13.6	9.5	22.3	11.3
Scenario 15						
LOCRM12	58.4	20.1	73.2	30.8	3.7	4.2
Cai	41.5	10.9	72.0	22.8	5.8	11.7
YY	63.4	14.4	78.1	19.5	2.9	10.7
Scenario 16						
LOCRM12	93.4	37.5	−	−	6.6	13.5
Cai	99.5	42.5	−	−	0.5	8.5
YY	98.5	39.3	−	−	1.5	11.7

LOCRM: local data-based continual reassessment method; Cai et al.: Cai; YY: Yuan and Yin; MTDC(s): maximum tolerated dose combination(s); OBDC(s): optimal biological dose combination(s); TDC(s): target dose combination(s).

Sel% means the selection percentages of MTDC(s), TDC(s), or overdoses. No. of pts means the number of patients treated at MTDC(s), TDC(s), or overdoses.

**Figure 1.**
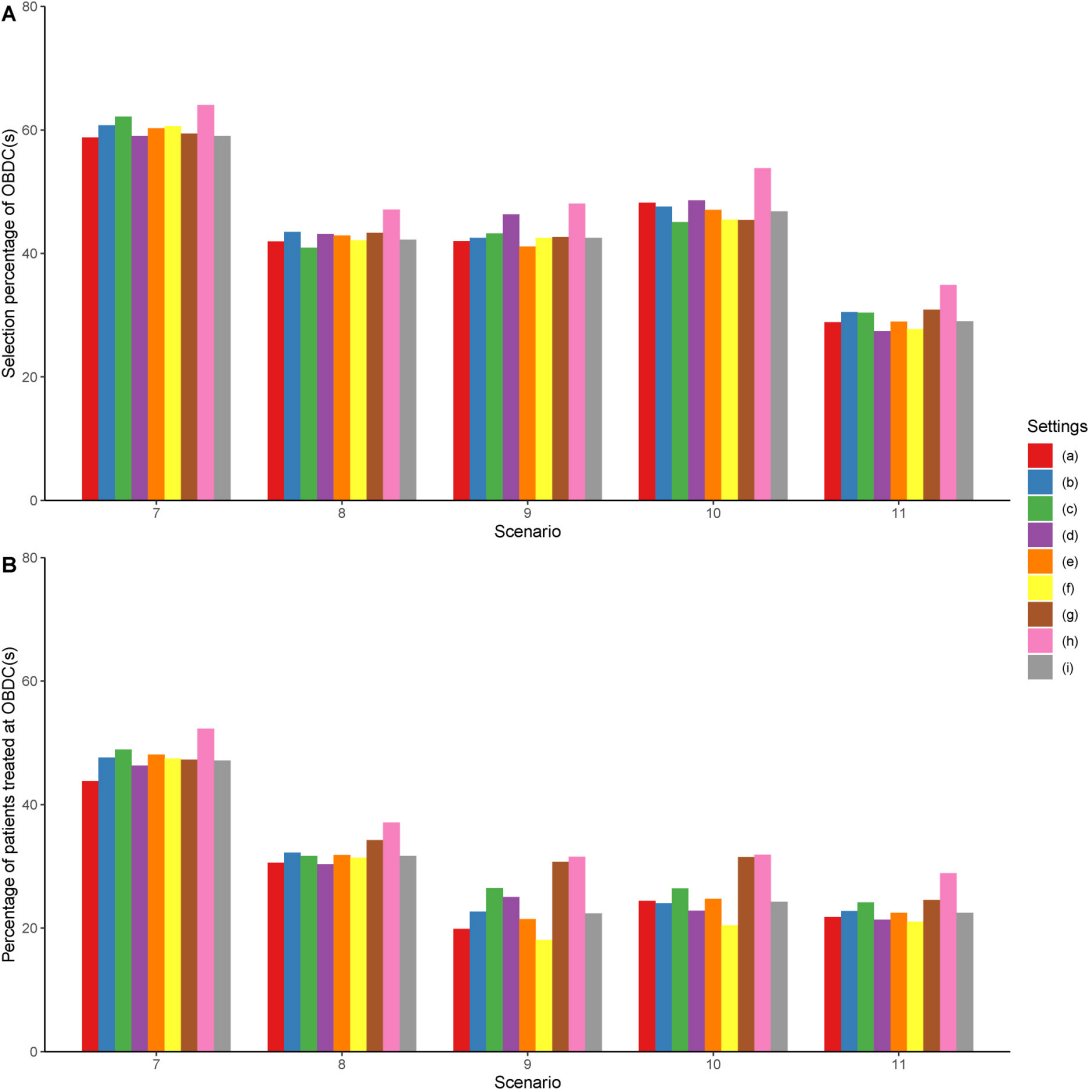
Results of the sensitivity analysis. Settings (a) to (h) shown in the legend correspond to the results under Settings (a) to (h) described in Section 5.3. Setting (i) corresponds to the results under the original setting described in Section 5.1.

### 5.3. Sensitivity analysis

In this section, we investigate the robustness of the proposed design under various configurations of the prior distributions or design parameters. Specifically, we study the impact of settings for toxicity modeling (i.e. the halfwidth of the indifference interval), the impact of parameters for efficacy modeling (including the hyperparameters α , $\beta_{1}$ , $\beta_{2}$ , $\gamma_{1}$ , and $\gamma_{2}$ ) and the value of z (which determines the aggressiveness in dose exploration as shown in Algorithm 1), and the impact of the sample size and cohort size as follows: Setting (a): the halfwidth of the indifference interval = 0.03; Setting (b): $a∼N(0,(\sqrt{1.34}{)}^{2})$ ; Setting (c): $\sigma_{\alpha }^{2}=\sigma_{\beta }^{2}=\sigma_{\gamma }^{2}=3$ ; Setting (d): z=1 ; Setting (e): z=3 ; Setting (f): incorporate five doses (i.e. set A ) in the efficacy model; Setting (g): Cohort size =1 ; Setting (h): Sample size =72 .

In Setting (a), we investigate the performance of LOCRM12 using a smaller halfwidth of the toxicity indifference interval. Technically, a smaller halfwidth indicates the distance between prior toxicity probabilities is smaller, which to some extent promotes escalation. In Setting (b), we use a smaller (more informative) prior variance for the unknown parameter used in the CRM model. In Setting (c), we similarly examine larger prior variances in the efficacy model. In Settings (d) and (e), we study the performance of LOCRM12 under different values of z , which controls the aggressiveness in dose exploration. In Setting (f), we investigate whether incorporating data from the smaller set of five doses in set A only is sufficient for the efficacy model. Settings (g) and (f) investigate the performance of the design under different cohort sizes and sample sizes, respecitvely. Simulation results of Scenarios 7 to 11 are shown in Figure 1. The proposed method is relatively robust under Settings (a) to (e), with a fluctuation in the correct selection percentage no > 6% and a fluctuation in the percentage of patients allocated to the OBDCs no > 7% under each scenario. This indicates that the proposed LOCRM12 is fairly robust to the considered configurations under Settings (a) to (e). By comparing (f) and (i) in Figure 1, it is observed that the design using A for the efficacy model is inferior to the design using B on average. As a result, incorporating more local data for efficacy monitoring may be more efficient and accurate in terms of finding the OBDCs. By comparing (g) and (i), we find that LOCRM12 is fairly robust to different cohort sizes, but a cohort size of three may slightly outperform a cohort size of one in most scenarios. A cohort size of three is generally preferred as using a cohort size of one may add additional complexity in implementation and logistics. Furthermore, according (h) and (i), LOCRM12 performs better as the sample size increases. As a result, if time and budget are not an issue, a larger sample size can improve the design’s accuracy of identifying the optimal dose combination.

## 6. Discussion

This article presents two designs, LOCRM and LOCRM12, for the early-phase exploratory dose-combination trials to determine the MTDC and OBDC, respectively. The LOCRM design uses CRM locally and makes escalation and de-escalation decisions among the adjacent doses of the current dose (i.e. A ). Local modeling and decision-making make LOCRM efficient and robust in various scenarios. Simulations show that LOCRM is comparable to the POCRM and $\mathrm{BOI}N_{\mathrm{Comb}}$ designs in identifying MTDC, and has a better safety profile than other methods due to the local model. The LOCRM12 design uses LOCRM as a toxicity model and a robit regression model as the efficacy model. The extra parameter (degrees of freedom) in the robit regression enhances the efficacy estimate’s robustness. Extensive simulations have shown that LOCRM12 performs better than other model-based methods in most considered scenarios. The simulation results also suggest that the proposed designs can perform well if the prior distributions are reasonable. If clinicians have prior knowledge of the investigational drug’s toxicity or efficacy, they can incorporate this information by setting appropriate informative priors. Setting appropriate prior distributions and design parameters is a nontrivial task for model-based designs. Although our designs use simpler models than many others, proper calibration of priors and parameters is still required. Effective collaboration between biostatisticians and clinicians is crucial to ensure the design’s efficiency and robustness. R codes for implementing the LOCRM and LOCRM12 designs are available at the GitHub repository https://github.com/ruitaolin/LOCRM.
